# Adenovirus pneumonia mimicking osimertinib‐induced pneumonitis in a patient with advanced NSCLC with *EGFR* mutation: A case report

**DOI:** 10.1111/1759-7714.15250

**Published:** 2024-02-21

**Authors:** Toru Imai, Tatsuya Yoshida, Yuichiro Ohe

**Affiliations:** ^1^ Department of Thoracic Oncology National Cancer Center Hospital Tokyo Japan

**Keywords:** adenovirus pneumonia, drug‐related pneumonitis, osimertinib

## Abstract

Drug‐related pneumonitis (DRP) caused by epidermal growth factor receptor (EGFR)‐tyrosine kinase inhibitors (TKIs) is a fatal adverse event in patients with *EGFR*‐mutant non‐small cell lung cancer (NSCLC). The diagnosis of DRP is based on radiological findings, the temporal association of presentation with the initiation of a systemic therapeutic agent, and the exclusion of other likely causes. Here we report a case in which severe adenoviral pneumonia mimicking DRP occurred during treatment with osimertinib, and osimertinib was successfully resumed after recovery from adenoviral pneumonia.

## Graphical abstract

(A) At initial hospitalization. (B) One month after osimertinib readministration.

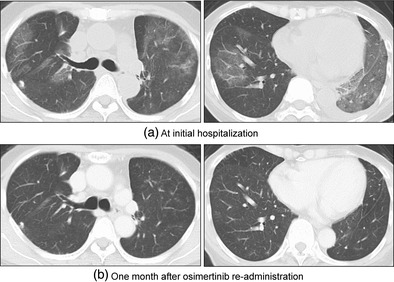

## INTRODUCTION

Osimertinib, a third‐generation epidermal growth factor receptor (EGFR)‐tyrosine kinase inhibitor (TKI), is the standard of care for treatment‐naïve advanced non‐small cell lung cancer (NSCLC) with *EGFR* mutations.^1^ However, EGFR‐TKIs are associated with side effects, primarily in the form of skin or gastrointestinal toxicities such as diarrhea.^2^ In general, Asian patients have a significantly higher risk of treatment‐related rash and diarrhea compared with Caucasian patients.^3^

Drug‐related pneumonia (DRP) caused by EGFR‐TKIs, including osimertinib, is a fatal adverse event in patients with *EGFR*‐mutant NSCLC.^4^ In the FLAURA study, the overall incidence of pneumonia ≥grade 3 during osimertinib treatment was 1%. However, Asians, especially Japanese patients, are more likely to develop EGFR‐TKI‐related pneumonitis.^5^ DRPs show various histological patterns and chest computed tomography (CT) findings.^6^ Chest CT patterns reflect the extent and distribution of lung abnormalities and have a prognostic value, but are not conclusive for diagnosis. The diagnosis of DRP is difficult, because it is based on radiological findings, the temporal association of presentation with the initiation of a systemic therapeutic agent, and the exclusion of other likely causes.

Here we report a case in which adenovirus pneumonia mimicking pneumonitis occurred during treatment with osimertinib, and osimertinib was successfully resumed after recovery from adenovirus pneumonia.

## CASE REPORT

**FIGURE 1:**
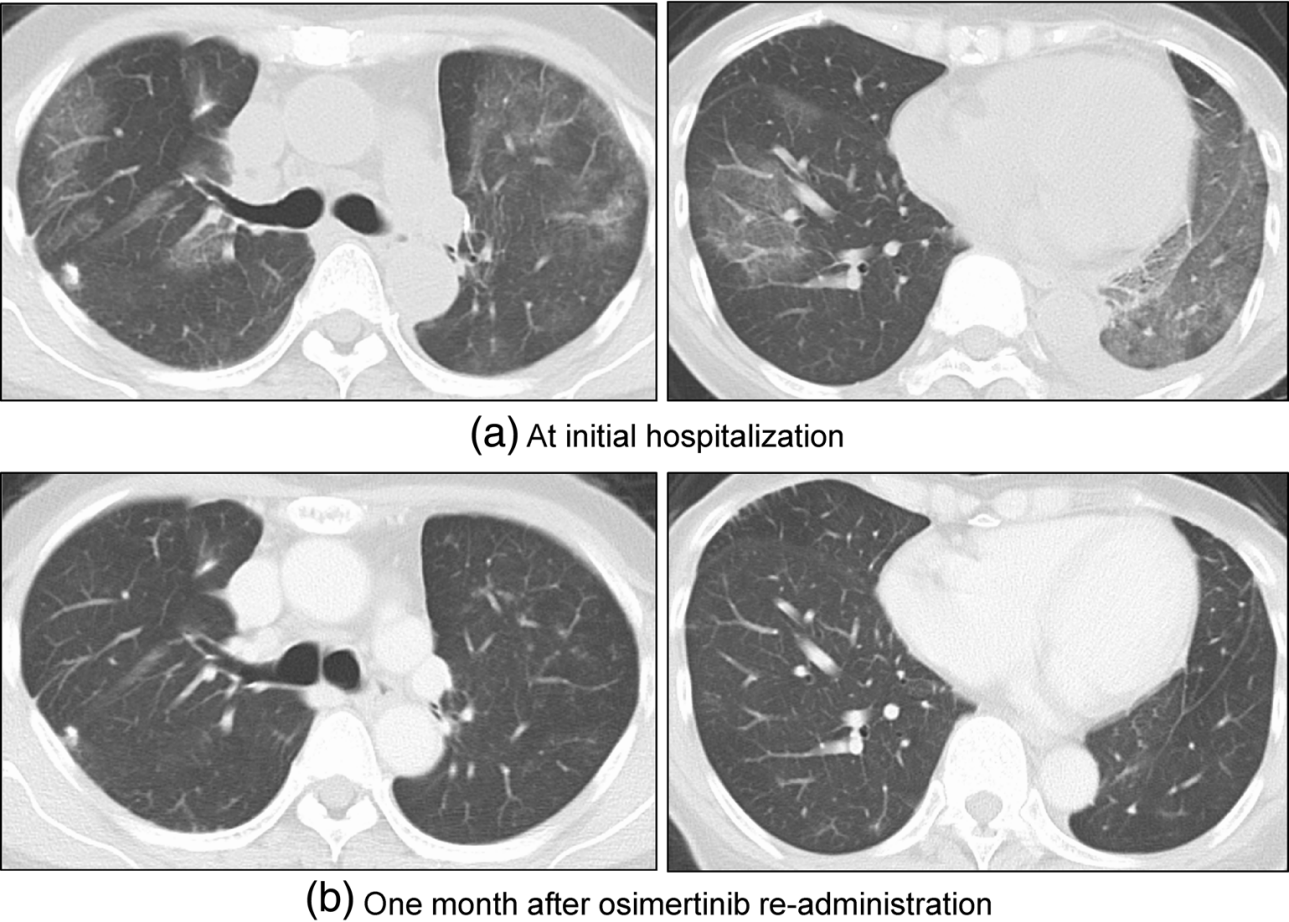
Chest computed tomography (CT) findings. (a) At the initial hospitalization for dyspnea, multiple ground‐glass opacities (GGOs) in both lung fields and new pleural effusion are observed. (b) One month after administration of osimertinib, the pleural effusion has resolved, and there is no evidence of interstitial lung disease recurrence.

**FIGURE 2:**
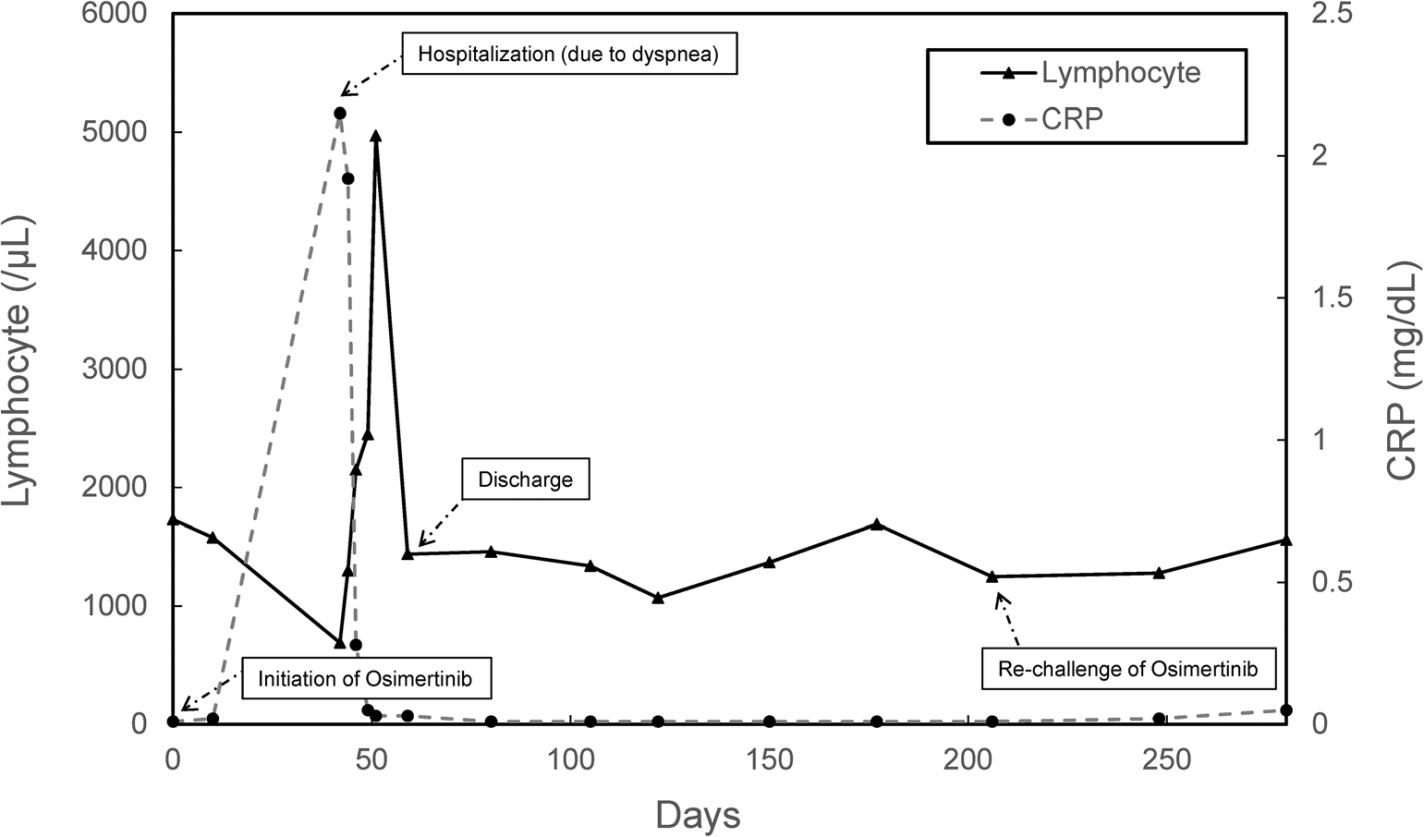
Treatment course (CRP and lymphocyte) from the first administration of osimertinib, through the episode of hospital admission and discharge for dyspnea, and up to the readministration of osimertinib.

A 69‐year‐old woman with no history of smoking developed postoperative recurrence of lung adenocarcinoma harboring an EGFR exon 19 deletion mutation. At baseline, she developed multiple bone metastases. The patient received osimertinib 80 mg/day as the initial systemic therapy. Six weeks after treatment initiation, the patient complained of dyspnea. Despite the absence of fever, the patient was admitted for supplemental oxygen therapy due to an oxygen saturation level of 90% on room air. Chest CT revealed multiple ground‐glass opacities (GGOs) in both lungs (Figure 1a). Initially, osimertinib‐induced pneumonitis was suspected, and prednisolone 1 mg/kg (50 mg/day) was initiated. Simultanously, multiplex polymerase chain reaction (PCR: FilmArray RP2.1 bioMérieux) testing for the detection of several viruses including the severe acute respiratory syndrome coronavirus‐2 was performed. The next day, it revealed adenovirus infection. After the initiation of prednisolone therapy, pneumonia and oxygenation levels improved within 1 week. After recovery from adenoviral pneumonia, osimertinib (80 mg/day) was restarted (Figure 1b). Pneumonia did not recur after readministration of osimertinib, and the patient was treated with osimertinib for more than 2 years. The changes in C‐reactive protein (CRP) levels and lymphocyte counts from the first administration of osimertinib, through the episode of hospital admission and discharge for dyspnea, and up to the readministration of osimertinib are shown in Figure 2.

## DISCUSSION

Chest CT plays an important role in the diagnosis and monitoring of DRP.^6^ Different radiographic patterns are observed in EGFR‐TKI‐related pneumonitis, including cryptogenic organizing pneumonia, hypersensitivity pneumonitis, nonspecific interstitial pneumonia, and diffuse alveolar damage (DAD)/acute interstitial pneumonia.^4^ Among these, DAD, characterized by diffuse or multifocal GGOs or consolidations with lung volume loss and traction bronchiectasis, is associated with high mortality. The treatment of pneumonitis during EGFR‐TKI therapy is mainly supportive and includes supplemental oxygen, empirical antibiotics, and systemic corticosteroid therapy with the discontinuation of EGFR‐TKIs.

Adenoviruses have been reported to cause pneumonia even in adults with normal immunity. Severe cases with respiratory failure may exhibit multifocal infiltrates, monocytopenia, and pleural effusions.^7^ Typical imaging features of adenovirus pneumonia, such as multifocal consolidation and GGOs, which are similar to those in DAD due to DRP, were observed in the present case.^8^ The imaging findings of viral pneumonia are variable and may overlap with those of nonviral pneumonia, such as that caused by DRP or inflammatory diseases. We attributed this event to osimertinib‐related pneumonitis. However, because of the unique circumstances of the coronavirus disease 2019 pandemic, we performed various tests including multiplex PCR using nasopharyngeal swabs, which revealed adenovirus infection. Multiplex PCR for detecting viral infections is rarely performed in patients treated with osimertinib when DRP is suspected. Therefore, in clinical practice, the number of patients with viral pneumonia inaccurately diagnosed with DRP remains unclear. Our case suggests the need to exclude viral pneumonia prior to the diagnosis of DRP based on not only the status of the immune system but also other infection parameters in terms of differential diagnosis.^9^, ^10^ Indeed, this patient resumed and successfully continued osimertinib 80 mg/day without DRP after recovery from adenoviral pneumonia. Further studies focusing on the clinical significance of testing for viral infections in suspected patients with DRP due to EGFR‐TKIs are needed.

## AUTHOR CONTRIBUTIONS

TI and TY made substantial contributions to the conception of the work. TI drafted the original manuscript. TY and YO reviewed the manuscript draft and revised it critically on intellectual content. All authors approved the final version of the manuscript to be published.

## CONFLICT OF INTEREST STATEMENT

The authors declare that they have no known competing financial interests or personal relationships that could have appeared to influence the work reported in this study.
